# A symptom network approach to schizophrenia in the CATIE study: processing speed as the central cognitive impairment

**DOI:** 10.1192/bjo.2025.10929

**Published:** 2026-01-20

**Authors:** Khan Buchwald, Richard J. Siegert, Matthieu Vignes, Ajit Narayanan, Margaret Sandham

**Affiliations:** School of Sciences, Auckland University of Technology, Auckland, New Zealand; Department of Psychology and Neuroscience, Auckland University of Technology, Auckland, New Zealand; School of Mathematical and Computational Sciences, Massey University, Palmerston North, New Zealand; Engineering, Computer, and Mathematical Sciences, Auckland University of Technology, Auckland, New Zealand; School of Psychology, Massey University, Auckland, New Zealand

**Keywords:** Schizophrenia, psychosis, cognition, Bayesian network, symptom network

## Abstract

**Background:**

People diagnosed with schizophrenia can have functional impairments in multiple domains. Cognitive impairment is central to schizophrenia and has substantial prognostic value compared with other symptoms of schizophrenia. However, no study has previously investigated directed relationships in a complex system of cognitive, sociodemographic, clinical and quality of life (QOL) variables in people diagnosed with schizophrenia.

**Aims:**

To identify the complex relationships of components of cognition with other cognitive components, as well as with clinical and QOL variables.

**Method:**

This study included data from 1450 participants in the Clinical Antipsychotic Trials of Intervention Effectiveness (CATIE) study. The present study reconstructed a Bayesian network from this data using cognition, clinical, sociodemographic and QOL variables.

**Results:**

Processing speed was centrally associated with all other cognitive domains. Cognitive domains were conditionally independent of positive symptoms but moderately associated with negative symptoms (*β* = −0.25; *P* < 0.001). The positive symptoms subscale was independent of QOL, conditioning on third variables. Negative symptoms were moderately associated with QOL (*β* = −0.33; *P* < 0.001), and processing speed had a weak association with QOL (*β* = −0.12; *P* < 0.001). Processing speed was a central variable in the network.

**Conclusions:**

Intervening with respect to processing speed may be the most beneficial way of improving other cognitive functions. More research is needed on directed networks that include social cognition and global levels of functioning.

Schizophrenia is a major mental health disorder that often leads to persistent cognitive, social and functional impairments and has the 12th highest disability weight of all conditions according to the Global Burden of Disease study.^1,2^ The importance of cognitive impairments in schizophrenia has been acknowledged since the time of Kraepelin^3^ and Bleuler,^4^ and recent research has clarified the centrality of cognitive dysfunction in the disorder.^5–7^ Despite the development of the Measurement and Treatment to Improve Cognition in Schizophrenia (MATRICS), no consensus exists on which cognitive abilities to target or prioritise in therapies aimed at enhancing cognition.^8^ Symptom network analysis is a promising new means of addressing this question. Symptom networks are graphical models that can be applied according to the network theory of mental disorders, which construes a psychological disorder as a complex, dynamic system of interacting symptoms. Symptom networks may help to elucidate the dependencies among different cognitive domains and other clinical or psychosocial variables and assist in clarifying treatment targets for cognitive remediation and psychopharmacology.

A growing body of research has included cognition in symptom networks of schizophrenia,^5,9–11^ and in our systematic review of symptom networks of schizophrenia and schizophreniform and schizoaffective disorders, cognition was a central component across all symptom network studies of schizophrenia that included cognition in the network.^5^ In particular, Abplanalp et al^11^ noted that processing speed was the primary predictor of other cognitive processes. Our recent systematic review identified a research gap, in that no published network studies have examined the directional associations of symptoms with cognitive functioning (or vice versa), other clinical correlates and quality of life (QOL) outcomes in individuals with schizophrenia.^5^ Here, we aimed to build on a strong body of previous studies that have identified the relationships between cognition and other domains of health, such as negative symptoms,^12–14^ or QOL.^15,16^ We used Bayesian networks for the added utility of being able to identify complex relationships, to help clarify which cognitive impairments had associations or conditional independencies with other cognitive functions, symptoms and QOL. Consequently, we hypothesise that cognitive indices are central to the network, and that cognitive variables are associated with QOL, general symptoms and negative symptoms, but not with positive symptoms.

## Method

### Participant characteristics

This study analysed data provided by 1450 people diagnosed with schizophrenia (PDS) from the National Institute of Mental Health Data Archive CATIE study. Participants recruited into this study were aged between 18 and 65 years, had a research diagnosis of schizophrenia and were able to take oral antipsychotic medication. Participants were excluded if they: (a) had a diagnosis of schizoaffective disorder, intellectual disability or other cognitive disorder; (b) had previously had severe adverse reactions to the proposed treatments; (c) had only had one psychotic episode; (d) had previously been treatment resistant; (e) were pregnant or breast-feeding; or (f) were considered to have a severe and unstable medical condition.^17^ We added no further exclusion criteria to this sample except for removing participants with more than half of their data missing. The authors assert that all procedures contributing to this work comply with the ethical standards of the relevant national and institutional committees on human experimentation and with the Helsinki Declaration of 1975, as revised in 2013. All procedures involving human participants and/or patients were approved by the Auckland University of Technology Ethics Committee (22/387 Static Symptom Networks of Schizophrenia) for the subsequent analyses of the CATIE study conducted in the current study. Written informed consent and ethical approval were provided for the original CATIE study.^17^ Table 1 provides the descriptive statistics for participants selected for the CATIE trial. A total of 1057 (74.4%) of the sample were males, with the average age of the sample being 40.5 years (s.d. = 11.1). Participants identified themselves as White or European American (871; 60.2%), Black or African American (504; 34.9%) or Asian American (33; 2.3%). Some participants identified with more than one race (26; 1.8%). In addition, 863 participants had never been married (59.6%) and 1216 did not work (84.9%), and the participants had an average of 11.5 years of education.

**Table 1:** Demographic characteristics

Demographic	Frequency (mean)	Percentage (s.d.)	Missing
Sex			29
Male	1057	74.4	
Female	364	25.6	
Age in years	(40.5)	(11.1)	2
Race			4
White (European American)	871	60.2	
Black (African American)	504	34.9	
Asian	33	2.3	
Native American/Alaska Natives	8	0.6	
Hawaiian/Pacific Islander	4	0.3	
More than one race	26	1.8	
Marital status			2
Never married	863	59.6	
Divorced	298	20.6	
Married	166	11.5	
Separated	87	6.0	
Widowed	34	2.3	
Years of education	(11.5)	(3.6)	10
Employment			17
Did not work	1216	84.9	
Part-time	120	8.4	
Full time	97	6.8	

### Sampling procedures

The CATIE study was a multisite randomised controlled trial conducted between January 2001 and December 2004 at 57 US sites, including university clinics, veterans’ affairs medication centres, mental health agencies, non-profit agencies, private practice centres and mixed sites. Whereas the CATIE study compared the effectiveness of medications over time, we selected only the baseline data for analysis in the present study.

### Measures and covariates

A range of assessment data was available for this study. We included demographic, clinical, QOL and cognitive variables for our study. Demographic variables included age, sex, race, education, employment and marital status. Regarding clinical assessments, the Positive and Negative Syndrome Scale (PANSS) developed by Kay et al^18^ can be considered the gold standard outcome measure for measuring symptoms in studies of the efficacy of schizophrenia treatment.^19^ The PANSS has fair internal consistency and excellent interrater reliability^20,21^ and shows strong correlations with other symptom assessments of schizophrenia.^21^ We also included the total score from the Calgary Depression Scale for Schizophrenia (CDSS). The CDSS has moderate to excellent reliability, high divergent validity and strong predictive validity, and correlates well with other measures of depression.^22^ The Drug Attitude Inventory (DAI)^23^ and Insight and Treatment Attitudes Questionnaire (ITAQ)^24^ were included to identify illness insight and attitudes towards medication adherence. Another scale used in this study was the Clinical Global Impressions (CGI) scale, which was modified to consider an index of severity for drug and alcohol use rated by a clinician.^25^ We also included the clinician global impression severity index in the subsequent analyses. The QOL assessment tool used in the CATIE study was derived from Heinrichs et al,^26^ and the assessment used to evaluate cognition was MATRICS. In the MATRICS version used during the CATIE trial, five cognitive domains were assessed: verbal, vigilance, processing speed, reasoning and working memory. These five domains were constructed on the basis of the assessments identified in Table 2. We initially included a measure of defined daily dose of antipsychotic medication. However, this was not a parent variable, as discussed below, and was therefore removed.

**Table 2:** Clinical characteristics

Variable	Mean	Median	s.d.	Minimum	Maximum	Missing
PANSS						
Positive	18.5	18	5.6	7	38	3
Negative	20.2	20	6.4	7	41	3
General	37.0	37	9.3	16	70	3
CDSS total	4.6	4	4.4	0	22	2
DAI total	5.0	6	3.9	−10	10	15
ITAQ total	18.2	21	5.0	0	22	8
CGI drug use	1.4	1	0.7	1	5	2
CGI alcohol use	1.5	1	0.7	1	5	2
CGI severity	4.0	4	0.9	1	7	5
QOL	2.7	2.7	1.1	0.25	5.9	10
MATRICS components						
Verbal						
HVLT: number of items recalled (trial 1)	4.7	5	1.7	0	11	60
HVLT: number of items recalled (trial 2)	6.5	6	2.1	0	12	61
HVLT: number of items recalled (trial 3)	7.5	8	2.5	0	12	60
Vigilance						
CPT: d-prime score: two-digit	2.3	2.4	1.1	−2.3	4.3	330
CPT: d-prime score: three-digit	1.7	1.8	1.0	−3	5.0	324
CPT: d-prime score: four-digit	1.0	0.9	0.8	−4.4	4.0	338
Processing speed						
COWAT: category F	9.8	10	4.1	0	24	58
COWAT: category A	7.9	8	3.7	0	21	59
COWAT: category S	10.0	10	4.3	0	25	59
COWAT: category animals	13.8	14	4.9	0	31	57
COWAT: category fruits	10.1	10	3.4	2	21	58
COWAT: category vegetables	8.6	8	3.4	0	25	57
WAIS-R: Digit Symbol Test	37.2	36	13.5	0	90	71
Grooved Pegboard: trial 1	11.7	12	3.9	0	22	79
Grooved Pegboard: trial 2	13.2	13	4.0	0	25	79
Reasoning						
WCST: number of perseverative errors	13.6	10	10.1	0	62	189
WCST: number of completed categories	2.1	2	1.6	0	6	192
WCST: additional consecutive cards in the final category	2.1	0	2.9	0	9	225
WISC-R: mazes	17.5	18	6.1	0	28	81
Working memory						
Letter–number sequencing	10.2	10	4.5	0	23	77
CTVWM: no delay	2.8	1	6.0	0	105	222
CTVWM: 5-s delay	28.4	22	19.3	0	128	222
CTVWM: 15-s delay	30.6	25	19.3	0	125	222

PANSS, Positive and Negative Syndrome Scale; CDSS, Calgary Depression Scale for Schizophrenia; DAI, Drug Attitude Inventory; ITAQ, Insight and Treatment Attitudes Questionnaire; CGI, Clinical Global Impressions; QOL, Quality of Life; MATRICS, Measurement and Treatment Research to Improve Cognition in Schizophrenia; HVLT, Hopkins Verbal Learning Test; CPT, Continuous Performance Test; COWAT, Controlled Oral Word Association Test; WAIS-R, Weschler Adult Intelligence Scale-Revised; WCST, Wisconsin Card Sorting Task; WISC-R, Weschler Intelligence Scale for Children – Revised; CTVWM, Computerised Test for Visuospatial Working Memory.

### Data diagnosis

We removed participants from this study if they had more than 50% missing information in all variables reported in our study (*n* = 10). The remaining missing data (2.4% of all observations) were imputed using the random forest imputation by Stekhoven & Bühlmann,^27^ which allows both categorical and continuous variables to be imputed. This reduced the sample size to 1450, and all results reported in this study are derived from this subset of individuals from the CATIE study. All assessment subscales were assumed to be continuous, except the CGI subscales. The MATRICS subscales reported herein are provided as standardised scores (mean = 0, s.d. = 1) as opposed to age-standardised scores, as provided by Keefe et al.^28^ All demographic variables were considered to be discrete, except for age, for the random forest imputation. The Bayesian network algorithm does not allow categorical variables to be daughter nodes of continuous variables in the comprehensive bnlearn R version 4.9.4 package we used.^29,30^ Hence, we treated age as a categorical variable (ages <30, 31–45, >45 years) following imputation to ensure all demographics were discrete variables. We also assumed that the CGI subscales were continuous in the Bayesian network despite being based on a Likert scale. No other transformations were implemented.

### Analytic strategies

Networks are comprised of nodes (variables) and edges (associations between variables). One graphical network approach, Bayesian networks, uses directed acyclic graphs to decompose the joint probability distribution of the variables.^29^ Directed relationships encode the dependency structure among a set of variables. Parent nodes have an edge directed away from the node towards a child node, which has an incoming edge. On a Windows PC, we used R version 4.3.2^31^ to analyse and implement the Bayesian networks in this study. To reconstruct relationship networks, we implemented a hybrid Bayesian network using the hill climbing method from R package bnlearn.^29^ We used a hybrid Bayesian network, which allowed both discrete and continuous variables to be included in the model, using a mixture of multinomial distributions for the parameter estimation of the discrete variables and normal distributions for the parameter estimation of the continuous variables. A blacklist of disallowed edges was automatically included when using the bnlearn package,^29^ so that discrete variables (demographics) could not be child nodes of continuous variables (clinical assessments). No other blacklists were specified when reconstructing the network. The hill climbing algorithm was initialised with an empty network; edges were then iteratively deleted or added, or the edge direction was reversed to locally optimise a network score, which reflected a fit to the data and was penalised for complexity. Although this algorithm identified a single set of orientations for parent and child relationships, other network structures may have fitted the data equally well but had different edge orientations.^29^ We did not estimate the Markov equivalence class, which is a set of partially directed acyclic graphs that fit the data equally well. A Markov equivalence class would have been helpful to identify but not confirm potential causal relationships. Hence, we refer to relationships in this study as associations as opposed to causal relationships.^29^ The direction of edges in a Markov equivalence class only needs to be specified when they are compelled, to avoid cycles, or form part of converging connections, as described by Scutari & Denis.^29^ We used the Bayesian information criterion as a network score criterion. We implemented another score-based algorithm in bnlearn, Tabu, which led to the same solution in the network structure as the hill climbing algorithm.^29^

Several other statistical methods were employed to investigate the Bayesian network and its properties; an overview of these can be found in the Supplementary Material available at https://doi.org/10.1192/bjo.2025.10929. We expanded a single Bayesian network by reconstructing an averaged network, that is, an amalgamation of many, possibly suboptimal networks, using bootstrapped versions of the data. There is evidence that averaging network models improves predictive validity.^29^ The network reconstructed on the complete data is referred to as a Bayesian network, whereas the Bayesian network based on bootstrapped samples was called the averaged Bayesian network; these were two networks with different structures and parameters. Structural equation models (SEMs) were implemented on the structure of the Bayesian network and the averaged Bayesian network; these were used to estimate regression coefficients and evaluate the fit of the model. We included the comparative fit index, Tucker–Lewis index, root mean squared error of approximation, Bayesian information criterion and Akaike information criterion. We also evaluated the fit of the modelling using a chi-squared test of model fit. Following this, we computed the centrality statistics betweenness, closeness and degree. Bayesian networks allow the user to query conditional probability relationships between variables. We thus queried the Bayesian network for the parent and child relationships of processing speed and QOL to compute the final results. The R code used for the analysis can be found in the Data Availability statement, and additional information on the analytic strategies can be found in the Supplementary Material.

## Results

Table 2 presents an overview of the baseline assessment scores for the assessments included before imputation. Participants in this study generally presented with average scores on the positive (50th–55th percentile), negative (40th–45th percentile) and general (45th–50th percentile) subscales of the PANSS.^18^ On average, participants scored 4.6 on the CDSS, corresponding to reporting symptoms that were between absent and mild on average across the sample and across items (mean = 0.51 for each item). On average, the sample had a positive attitude towards medication, as indicated by the DAI. Similarly, participants on average responded with scores on the ITAQ indicating partial to good insight (mean = 1.7 for each item). For both CGI drug use and CGI alcohol use, scores indicated responses that were on average between abstinence and substance use without impairment. The QOL scale mean scores reflected intermediate but significant levels of impairment.

Regarding the MATRICS cognitive battery, most participants scored between −1.6 standard deviations and −2.5 standard deviations from the mean on the Hopkins Verbal Learning Test, based on the results from the CATIE trial.^28^ Participants also performed 1.5 standard deviations or below the mean for males across all ages for the three indices of the Identical Pairs Continuous Performance Test, based on the norms published by Rapisarda et al,^32^ and between the 9th and 10th percentiles for processing speed and the Controlled Oral Word Association Test, based on the norms of Ruff et al.^33^ Last, the participants in this study scored 2.1 standard deviations below the mean for letter number sequencing based on the norms of Gold et al.^34^

Table 3 shows the fit statistics for each model and the significance of the implied variance–covariance matrix and observed variance–covariance matrix. The averaged Bayesian network had considerably worse comparative fit index and Tucker–Lewis index than the Bayesian network; the root mean squared error of approximation was lower for the Bayesian network, and the Bayesian information criterion and Akaike information criterion favoured the Bayesian network compared with the averaged Bayesian network. Both models were significant (*P* < 0.001), indicating that the model-implied variance–covariance matrix significantly differed from the observed variance–covariance matrix in both models. Given that the Bayesian network had a better fit than the averaged Bayesian network, subsequent results were based on the Bayesian network. Furthermore, many of the relationships in the averaged Bayesian network were also found in the Bayesian network; 29 edges were shared between the two networks, whereas 18 edges were unique to the Bayesian network, and 16 edges were unique to the averaged Bayesian network. The network plot, SEM plot, centrality statistics and adjusted *P*-values for the averaged Bayesian network can be found in Supplementary Figs 1–3 and Supplementary Table 2.

**Table 3:** Fit statistics for the Bayesian network and averaged Bayesian network

Model	CFI	TLI	RMSEA	*χ* ^2^	d.f.	*P*	BIC	AIC
Bayesian network	0.934	0.935	0.048	680.031	157	<0.001	65979.34	65261.36
Averaged Bayesian network	0.919	0.920	0.055	843.975	156	<0.001	66040.07	65411.83

CFI, Comparative Fit Index; TLI, Tucker–Lewis index; RMSEA, root mean square error of approximation; BIC, Bayesian information criterion; AIC, Akaike information criterion.

**Fig. 1:**
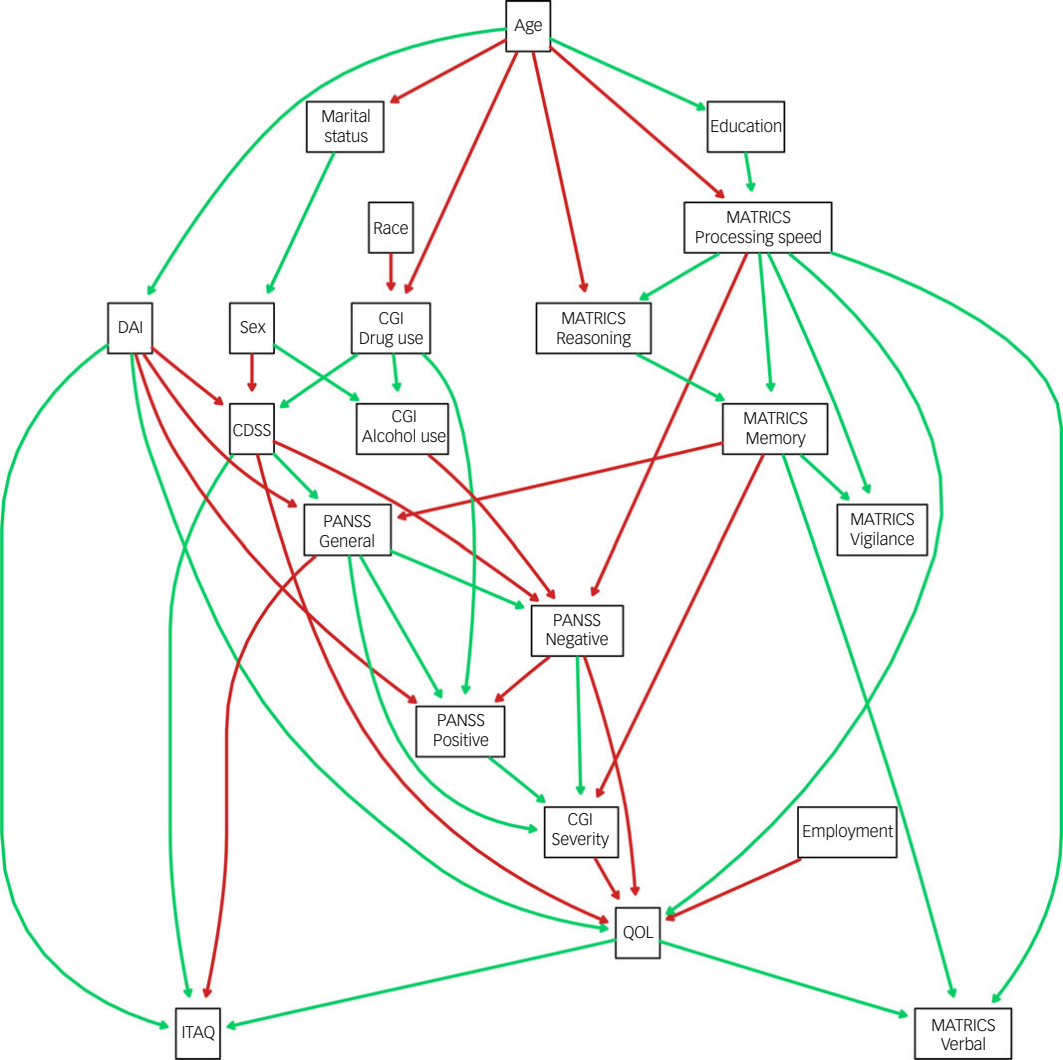
Averaged Bayesian network. MATRICS, Measurement and Treatment Research to Improve Cognition in Schizophrenia; DAI, Drug Attitude Inventory; CDSS, Calgary Depression Scale for Schizophrenia; CGI, Clinical Global Impression; PANSS, Positive and Negative Syndrome Scale; QOL, Quality of Life; ITAQ, Insight and Treatment Attitudes Questionnaire.

Figure 1 shows the structure of the reconstructed Bayesian network. Edges were coloured in green if the estimated regression coefficient was positive in the SEM and red if the coefficient was negative. These coefficients took into consideration the effects of other confounding variables. Age, employment and race were not child nodes of any other variables, whereas the MATRICS vigilance subscale, MATRICS verbal subscale and ITAQ total score were child nodes only. The MATRICS processing speed was associated with all other MATRICS scales (positive relationship). Moreover, MATRICS reasoning was conditionally independent of MATRICS vigilance, given the values for MATRICS working memory. The PANSS general subscale was associated with MATRICS working memory (a negative relationship, as inferred by the SEM fit), and the PANSS negative subscale was associated with MATRICS processing speed (negative relationship). The PANSS negative subscale was conditionally independent of all other MATRICS subscales, given scores on processing speed. The PANSS positive subscale was conditionally independent of cognition as assessed by the MATRICS and conditionally independent of the MATRICS given either the PANSS negative subscale or the PANSS general subscale. QOL was associated with the DAI total score (positive relationship), CDSS total score (negative relationship), CGI severity index (negative relationship), PANSS negative subscale (negative relationship), MATRICS processing speed (positive correlation) and employment (negative relationship). QOL was conditionally independent of age, education, sex, MATRICS working memory, PANSS positive subscale, PANSS general, CGI drug use and CGI alcohol use, given the parents’ DAI total, CDSS total, CGI severity index, PANSS negative subscale, and MATRICS processing speed. CGI drug use index was associated with the CDSS total (positive relationship), and the PANSS positive subscale (positive relationship) was associated with CGI drug use. The CGI alcohol use index was a parent of the PANSS negative subscale (negative relationship), and the PANSS positive subscale was conditionally independent of CGI alcohol use given the PANSS negative subscale.

**Fig. 2:**
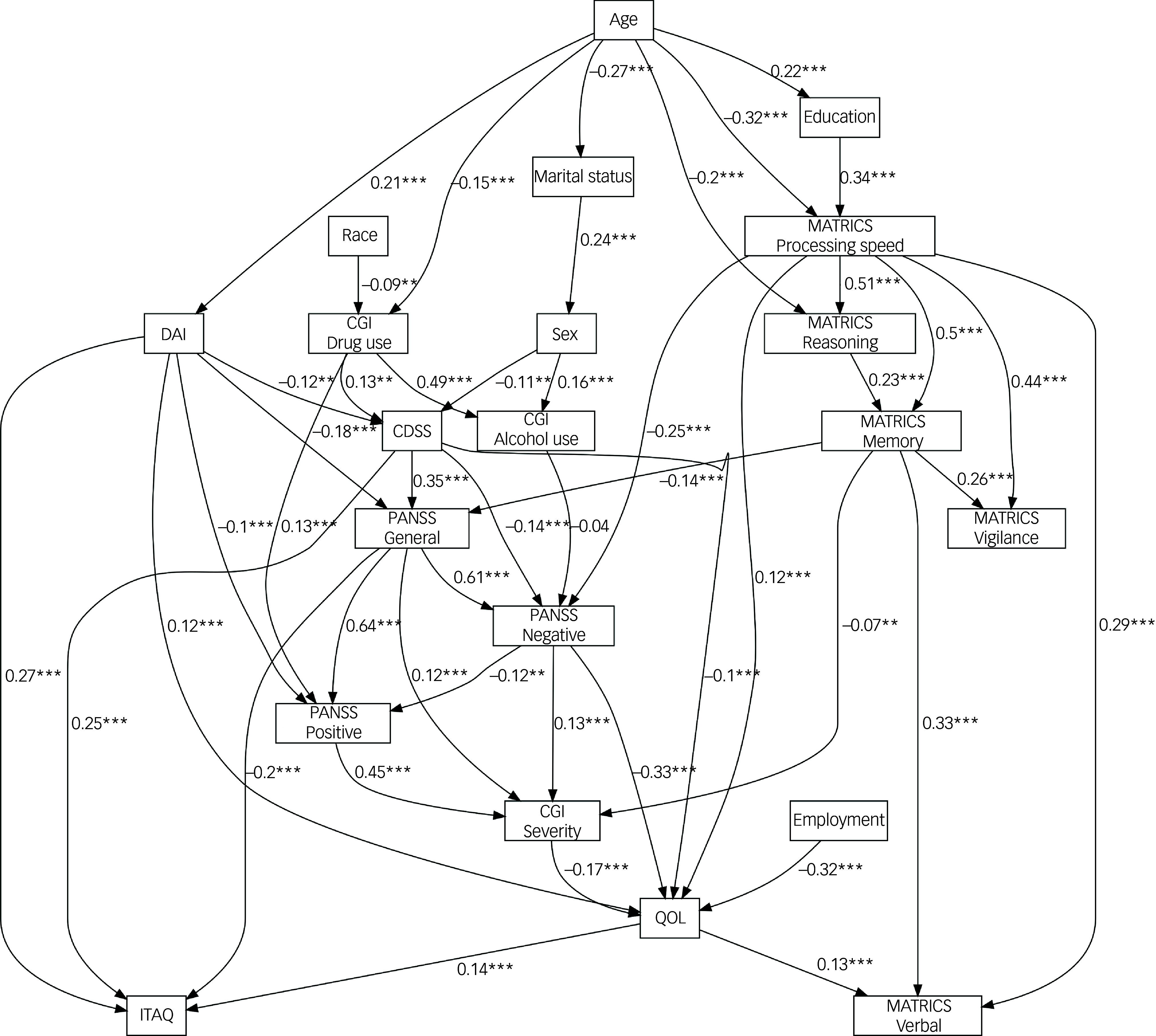
Structural equation model of averaged Bayesian network. MATRICS, Measurement and Treatment Research to Improve Cognition in Schizophrenia; DAI, Drug Attitude Inventory; CDSS, Calgary Depression Scale for Schizophrenia; CGI, Clinical Global Impression; PANSS, Positive and Negative Syndrome Scale; QOL, Quality of Life; ITAQ, Insight and Treatment Attitudes Questionnaire. ** *p* < .01; *** *p* < .001.

Figure 2 shows the same network structure as Fig. 1 but with the addition of standardised regression coefficients and significance values for the relationships between parent and child variables. All *P*-values were subject to the multiple comparison correction method of Holm,^35^ and all Holm-adjusted *P*-values can be found in Supplementary Table 1. All the relationships between the MATRICS subscales were positive and significant, with the strongest relationships being those between the MATRICS processing speed scale and the MATRICS vigilance and MATRICS working memory subscales. The MATRICS processing speed subscale had a significant and moderate negative association with the PANSS negative subscale (*b* = −0.25; *P* < 0.001), and the MATRICS memory subscale was a negative and significant moderate parent of the PANSS general subscale (*b* = −0.14; *P* < 0.001). The PANSS general subscale had a strong significant positive relationship with the PANSS negative subscale (*b* = 0.61; *P* < 0.001) and PANSS positive subscale (*b* = 0.64; *P* < 0.001). There was also a strong positive relationship between the CDSS and PANSS general (*b* = 0.35; *P* < 0.001), but the CDSS had a moderate, negative and significant association with the PANSS negative subscale (*b* = −0.14; *P* < 0.001). The PANSS positive subscale had a strong positive and significant association with the CGI severity index (*b* = 0.45; *P* < 0.001). Of the six parents of QOL, the strongest association was that for the PANSS negative subscale, which was significant and negatively associated (*b* = −0.33; *P* < 0.001) with QOL scores. Last, the MATRICS processing speed subscale had a moderate and significant association with QOL (*b* = 0.12; *P* < 0.001), and employment was also associated with QOL, via a strong negative relationship (*b* = −0.32; *P* < 0.001). The parents of QOL explained a total of 37.1% of the variance in QOL.

**Fig. 3:**
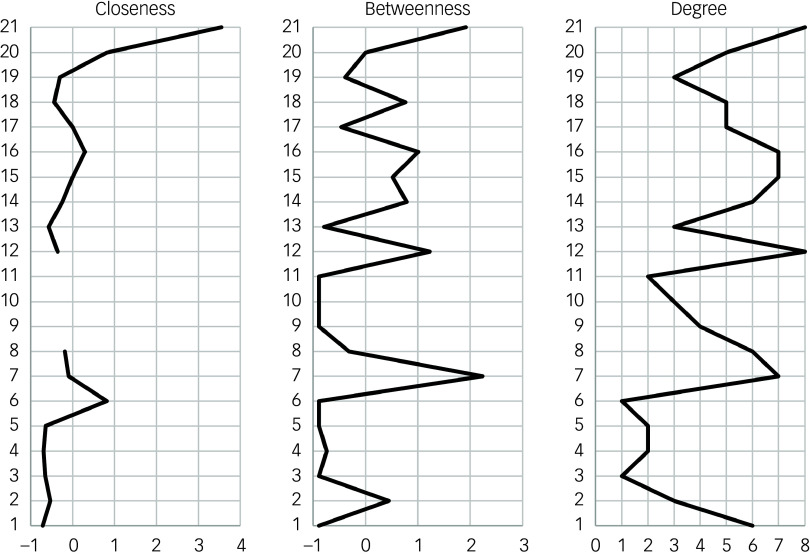
Centrality statistics of the averaged Bayesian network. Standardised values are given for closeness and betweenness: 1, age; 2, sex; 3, race; 4, marital status; 5, education; 6, employment; 7, Calgary Depression Scale for Schizophrenia total; 8, Drug Attitude Inventory total; 9, Insight and Treatment Attitudes Questionnaire total; 10, Measurement and Treatment Research to Improve Cognition in Schizophrenia (MATRICS) verbal; 11, MATRICS vigilance; 12; MATRICS processing speed; 13, MATRICS reasoning; 14, MATRICS working memory; 15, Positive and Negative Syndrome Scale (PANSS) general; 16, PANSS negative; 17, PANSS positive; 18, Clinical Global Impression (CGI) drug use; 19, CGI alcohol use; 20, CGI severity; 21, quality of life.

The three charts in Fig. 3 show the centrality statistics for the Bayesian network. QOL had the highest closeness, followed by employment and CGI severity index. The CDSS had the highest betweenness, followed by QOL and the MATRICS processing speed subscale. For degree, QOL and the MATRICS processing speed subscale had the highest numbers of edges (eight), followed by the CDSS, PANSS negative subscale and PANSS general subscale with seven edges each. The MATRICS processing speed subscale had more outgoing edges than QOL.

Table 4 presents the probability queries on the network’s MATRICS subscales and variables associated with these subscales. All values in the table are based on the median response for that variable. Overall, the results suggested that the associations between MATRICS subscales were stronger than those between MATRICS nodes and nodes representing the PANSS negative subscale, PANSS positive subscale, CGI severity and QOL, for median values. For the largest conditional associations, the probability of an individual scoring higher than the median on QOL, given that they were employed, was 0.795. In addition, the probability of a participant scoring less than the median on MATRICS memory was 0.767 if they scored less than the median for MATRICS processing speed and 0.724 if they scored less than the median for MATRICS reasoning if they scored less than the median for MATRICS processing speed. Scoring above the median on the PANSS negative was also associated with a 0.632 probability of scoring less than the median on the QOL index, and scoring below the median of processing speed was associated with a 0.452 probability of scoring above the median on the QOL index.

**Table 4:** Network conditional probability queries of MATRICS subscales and parents of QOL

Variable 1	Sign^a^	Median value	Variable 2	Sign^a^	Median value	Probability^b^
MATRICS processing speed	<	−0.03	MATRICS reasoning	<	0.03	0.685
MATRICS processing speed	<	−0.03	MATRICS memory	<	0.13	0.767
MATRICS processing speed	<	−0.03	MATRICS vigilance	<	−0.07	0.668
MATRICS processing speed	<	−0.03	MATRICS verbal	<	−0.04	0.659
MATRICS processing speed	<	−0.03	PANSS negative	>	−0.03	0.589
MATRICS processing speed	<	−0.03	QOL	>	−0.09	0.452
MATRICS reasoning	<	0.03	MATRICS memory	<	0.13	0.724
MATRICS memory	<	0.13	MATRICS vigilance	<	−0.07	0.624
MATRICS memory	<	0.13	MATRICS verbal	<	−0.04	0.641
MATRICS memory	<	0.13	PANSS general	>	0	0.558
MATRICS memory	<	0.13	CGI severity	>	0.04	0.526
DAI	<	0	QOL	>	−0.09	0.489
CGI severity	>	0.04	QOL	>	−0.09	0.424
PANSS negative	>	−0.03	QOL	>	−0.09	0.381
CDSS	>	−0.13	QOL	>	−0.09	0.478
Employment	=	Full-time	QOL	>	−0.09	0.795
Employment	=	Part-time	QOL	>	−0.09	0.704
Employment	=	Did not work	QOL	>	−0.09	0.497

MATRICS, Measurement and Treatment Research to Improve Cognition in Schizophrenia; QOL, Quality of Life; PANSS, Positive and Negative Syndrome Scale; CGI, Clinical Global Impression; DAI, Drug Attitude Inventory; CDSS, Calgary Depression Scale for Schizophrenia.

a. ‘Sign’ indicates whether scores are less than (<), greater than (>) or equal to (=) the median value.

b. ‘Probability’ indicates the probability of variable 2 being less than or greater than the median value, if variable 1 is less than or greater than the median.

## Discussion

This study explored the directed relationships between cognitive, sociodemographic and clinical characteristics of PDS. We found that slower processing speed could be the primary cognitive impairment in PDS, as it was centrally located and associated with performance on all cognitive domains measured by the MATRICS. Processing speed was also associated with reduced subjective well-being (QOL) when we controlled for negative symptoms, illness severity and depression. Furthermore, cognitive performance was not associated with positive symptoms of schizophrenia; however, slower processing speed was related to greater severity of negative symptoms. In addition, poorer working memory, as measured by the MATRICS, was associated with higher levels of general symptoms on the PANSS. For PDS, being in full-time employment was a strong protective factor that increased the probability of having a higher QOL.

Our findings were generally in agreement with those of other research. General research on the MATRICS has found that the seven-factor structure of the MATRICS yields the best model fit when correlations are allowed between the factors.^36^ Hence, the factors should be treated as non-orthogonal.^36^ Our findings support this by indicating that the MATRICS subscales are interdependent and not independent of one another. In particular, processing speed was centrally associated with all other MATRICS subscales, consistent with findings by other authors.^11^

The present study’s findings that processing speed had high network centrality, closeness and degree were also aligned with previous research.^5^ Abplanalp et al^11^ reported that processing speed was associated with all other MATRICS subscales and identified processing speed as a key cognitive process to target for treatment. We extended this research to identify other clinical correlates and QOL, fitted an SEM with standardised coefficients for effect sizes, and conducted probability queries on the network. A previous meta-analysis^37^ also found that processing speed was the most impaired of all cognitive domains in PDS; it also supported the order of impairment levels for the MATRICS subscales in PDS found in the present study, with the exception of vigilance.^37^

Research to date has not conclusively identified processing speed as most important for PDS, and there is no scientific or clinical consensus yet on which cognitive functions should be prioritised for the treatment of cognition for PDS.^8^ Working memory may also be a core cognitive deficit of schizophrenia, as it is associated with difficulties in handling social and interpersonal situations,^38^ and Halverson, Orleans-Pobee^39^ identified that social cognition was most important in explaining functional outcome. The CATIE data used in the present study predated the inclusion of social cognition as a subscale in the MATRICS. Future research could explore the centrality of processing speed found in the present study and that of Abplanalp et al^11^ and the findings of Halverson and Orleans-Pobee^39^ of social cognition and functional outcomes by including functional outcomes alongside the MATRICS subscales and symptom measures, using a Bayesian symptom network.

Processing speed had a significant positive relationship with QOL in our study, albeit with a small effect size (*β* = 0.12). In addition, greater severity of negative symptoms had the highest coefficient for association with QOL; this was supported by previous systematic reviews and a meta-analysis.^38,39^ Our findings were also consistent with other research suggesting that positive symptoms are conditionally independent of QOL when results are corrected for the association of negative symptoms, and with previous studies that reported negative symptoms being more associated with decreases in QOL compared with positive symptoms.^40,41^ The present study also identified full-time employment as a strong protective factor for PDS, compared with part-time employment or unemployment. Other research has reported that employment is associated with QOL.^42^ However, in contrast to Bouwmans et al,^42^ we did not find cognitive impairment and negative symptoms to be associated with employment in our study.

Processing speed from the MATRICS subscales was moderately associated with negative symptoms, in agreement with strong evidence from three systematic reviews and a combined meta-analysis.^12–14^ All three systematic reviews found a significant but modest negative association between negative symptoms and cognition. Notably, Veerman et al^43^ found that processing speed may mediate the association between working memory and negative symptoms. According to Melillo et al,^44^ processing speed may be more consistently associated with severity of negative symptoms compared with other cognitive functions. In our study, processing speed seemed to mediate the relationship between cognitive functioning and negative symptoms, as all other MATRICS subscales were conditionally independent of negative symptoms once we had accounted for processing speed.

Both the negative and the general subscales of the PANSS were identified as mediating variables in the present study, making cognitive assessment scores and the PANSS positive subscale conditionally independent. Although this aligned with previous evidence that cognition is weakly correlated with positive symptoms,^45^ we did not find this association in our network, and other evidence suggests independence between these symptom groups, as concluded in a meta-analysis.^46^ Galderisi et al^9^ found in their network study of schizophrenia that positive symptoms were not highly linked with other nodes in the network; however, our results suggested that they were was significantly associated with other PANSS subscales, as well as with drug attitude inventories and CGI severity. A later study by Galderisi et al^10^ found that positive symptoms were only weakly associated with other symptoms, whereas cognition was strongly associated with functional capacity. Amore et al^47^ used partial correlation networks of the PANSS, CDSS, MATRICS and other assessments. Although they did not comment on cognition specifically, they found that an index representing MATRICS scores was a central variable within the network.

The findings of the present study suggest that targeting processing speed in therapy for PDS could be more beneficial than focusing on other cognitive functions, as all other cognitive processes have associations with processing speed. Future research could identify plausible causal relationships between processing speed and other cognitive functions in PDS. Consistent with previous research,^36^ we found that processing speed was the most impaired cognitive function in PDS and may be the core cognitive deficit. Cognitive remediation for processing speed may globally improve cognitive processes, enhance QOL and reduce negative symptoms.^48^ However, treating this cognitive process in isolation may also be difficult and not ideal for PDS. Treatments may provide improvements across a range of cognitive functions,^49^ and a literature review has concluded that treating a specific cognitive function with cognitive remediation has a general effect rather than an isolated effect.^50^ Our results supported this concept, as we found that cognitive functions measured by the MATRICS had complex associations with each other. We conclude that processing speed may be the central cognitive impairment in schizophrenia, in agreement with Abplanalp et al.^11^

### Limitations

This study advances our understanding of the associations between variables by accounting for key confounders and modelling their interrelationships. However, several limitations should be acknowledged. First, the cross-sectional design precluded any complete inference of causal directionality. Network edges capture conditional associations, but these may reflect unmeasured or latent variables, which were not directly modelled and could substantially alter the observed structure.^48^ Second, although Bayesian networks provide a flexible framework for representation of relationships in the data, the approximation algorithms needed for model estimation did not allow us to rule out alternative structures that might explain the observed dependencies equally well or better.^48^ Third, orientation of edges should be interpreted with caution: when directionality is not compelled by the data, edges are retained irrespective of orientation, meaning that apparent causal interpretations (e.g. between processing speed and working memory) are not empirically justified. Another key consideration is that the measures used in the present study were from the CATIE pharmaceutical trial baseline assessment. Since that time, the conceptualisation of negative symptoms has slightly changed; consequently, the measures may have limited validity. Future studies should consider using the Clinical Assessment Interview for Negative Symptoms and the Brief Negative Symptom Scale for improved reliability and validity.^19^ Finally, the interpretation of centrality indices remains debated. Although they are commonly reported, their validity as indicators of intervention targets in psychological and cognitive networks is exploratory in nature, and any conclusions drawn from them should be validated empirically in future studies.^51^

## Supporting information

Buchwald et al. supplementary material Buchwald et al. supplementary material

## Data Availability

The data are available from the National Institute of Health Data Archive and are therefore not disseminated by the authors (NDA Study DOI: 10.15154/ev0h-1q75). The computer code to replicate the study is available in the link below. https://github.com/KhanBuchwald/Symptom_network_insights_from_the_CATIE_study_on_schizophrenia-The_impact_of_processing_speed.git.

## Supplementary material

The supplementary material is available online at https://doi.org/10.1192/bjo.2025.10929

## References

[1] Salomon JA , Haagsma JA , Davis A , de Noordhout CM , Polinder S , Havelaar AH , et al. Disability weights for the Global Burden of Disease 2013 study. Lancet Glob Health 2015; 3: e712–23. 26475018 10.1016/S2214-109X(15)00069-8

[2] Charlson FJ , Ferrari AJ , Santomauro DF , Diminic S , Stockings E , Scott JG , et al. Global epidemiology and burden of schizophrenia: findings from the global burden of disease study 2016. Schizophr Bull 2018; 44: 1195–203. 29762765 10.1093/schbul/sby058 PMC6192504

[3] Kraepelin E. Psychiatrie: Ein Lehrbuch für Studierende und Ärzte [*Psychiatry: A Textbook for Students and Doctors*] 8th ed. Verlag von Johann Ambrosius Barth, 1913.

[4] Bleuler E. Die Prognose der Dementia praecox (Schizophreniegruppe) [The prognosis of dementia praecox (schizophrenia group)]. Allgem Z Psychiatr psychisch-gerichtliche Medizin 1908; 31: 436–80.

[5] Buchwald K , Narayanan A , Siegert RJ , Vignes M , Arrowsmith K , Sandham M. Centrality statistics of symptom networks of schizophrenia: a systematic review. Psychol Med 2024; 54: 1061–73. 38174555 10.1017/S003329172300363X

[6] McCutcheon RA , Keefe RSE , McGuire PK. Cognitive impairment in schizophrenia: aetiology, pathophysiology, and treatment. Mol Psychiatry 2023; 28: 1902–18. 36690793 10.1038/s41380-023-01949-9 PMC10575791

[7] Gebreegziabhere Y , Habatmu K , Mihretu A , Cella M , Alem A. Cognitive impairment in people with schizophrenia: an umbrella review. Eur Arch Psychiatry Clin Neurosci 2022; 272: 1139–55. 35633394 10.1007/s00406-022-01416-6 PMC9508017

[8] Best MW , Bowie CR. A review of cognitive remediation approaches for schizophrenia: from top-down to bottom-up, brain training to psychotherapy. Expert Rev Neurother 2017; 17: 713–23. 28511562 10.1080/14737175.2017.1331128

[9] Galderisi S , Rucci P , Kirkpatrick B , Mucci A , Gibertoni D , Rocca P , et al. Interplay among psychopathologic variables, personal resources, context-related factors, and real-life functioning in individuals with schizophrenia: a network analysis. JAMA Psychiatry 2018; 75: 396–404. 29450447 10.1001/jamapsychiatry.2017.4607 PMC5875306

[10] Galderisi S , Rucci P , Mucci A , Rossi A , Rocca P , Bertolino A , et al. The interplay among psychopathology, personal resources, context-related factors and real-life functioning in schizophrenia: stability in relationships after 4 years and differences in network structure between recovered and non-recovered patients. World Psychiatry 2020; 19: 81–91. 31922687 10.1002/wps.20700 PMC6953544

[11] Abplanalp SJ , Lee J , Horan WP , Kern RS , Penn DL , Green MF. A Bayesian network approach to social and nonsocial cognition in schizophrenia: are some domains more fundamental than others? Schizophr Bull 2023; 49: 997–1006. 36869810 10.1093/schbul/sbad012 PMC10318874

[12] Dominguez Mde G , Viechtbauer W , Simons CJ , van Os J , Krabbendam L. Are psychotic psychopathology and neurocognition orthogonal? A systematic review of their associations. Psychol Bull 2009; 135: 157–71. 19210058 10.1037/a0014415

[13] Au-Yeung C , Penney D , Rae J , Carling H , Lassman L , Lepage M. The relationship between negative symptoms and MATRICS neurocognitive domains: a meta-analysis and systematic review. Progr Neuro-Psychopharmacol Biol Psychiatry 2023; 127: 110833. 10.1016/j.pnpbp.2023.110833 37482283

[14] Melillo A , Caporusso E , Giordano GM , Giuliani L , Pezzella P , Perrottelli A , et al. Correlations between negative symptoms and cognitive deficits in individuals at first psychotic episode or at high risk of psychosis: a systematic review. J Clin Med 2023; 12: 7095. 38002707 10.3390/jcm12227095 PMC10672428

[15] Dong M , Lu L , Zhang L , Zhang Y-S , Ng CH , Ungvari GS , et al. Quality of life in schizophrenia: a meta-analysis of comparative studies. Psychiatr Quart 2019; 90: 519–32. 10.1007/s11126-019-09633-4 31119453

[16] Marder SR , Galderisi S. The current conceptualization of negative symptoms in schizophrenia. World Psychiatry 2017; 16: 14–24. 28127915 10.1002/wps.20385 PMC5269507

[17] Swartz MS , Stroup TS , McEvoy JP , Davis SM , Rosenheck RA , Keefe RS , et al. Special section on implications of CATIE: what CATIE found: results from the schizophrenia trial. Psychiatr Serv 2008; 59: 500–6. 18451005 10.1176/ps.2008.59.5.500 PMC5033643

[18] Kay SR , Fiszbein A , Opler LA. The Positive and Negative Syndrome Scale (PANSS) for schizophrenia. Schizophr Bull 1987; 13: 261–76. 3616518 10.1093/schbul/13.2.261

[19] Kumari S , Malik M , Florival C , Manalai P , Sonje S. An assessment of five (PANSS, SAPS, SANS, NSA-16, CGI-SCH) commonly used symptoms rating scales in schizophrenia and comparison to newer scales (CAINS, BNSS). J Addict Res Ther 2017; 8: 324. 29430333 10.4172/2155-6105.1000324 PMC5805140

[20] Peralta V , Cuesta MJ. Psychometric properties of the positive and negative syndrome scale (PANSS) in schizophrenia. Psychiatry Res 1994; 53: 31–40. 7991730 10.1016/0165-1781(94)90093-0

[21] Bell M , Milstein R , Beam-Goulet J , Lysaker P , Cicchetti D. The Positive and Negative Syndrome Scale and the Brief Psychiatric Rating Scale. Reliability, comparability, and predictive validity. J Nerv Ment Dis 1992; 180: 723–8. 1431824 10.1097/00005053-199211000-00007

[22] Lako IM , Bruggeman R , Knegtering H , Wiersma D , Schoevers R , Slooff C , et al. A systematic review of instruments to measure depressive symptoms in patients with schizophrenia. J Affect Disord 2012; 140: 38–47. 22099566 10.1016/j.jad.2011.10.014

[23] Awad AG. Subjective response to neuroleptics in schizophrenia. Schizophr Bull 1993; 19: 609–18. 7901897 10.1093/schbul/19.3.609

[24] McEvoy JP , Apperson LJ , Appelbaum PS , Ortlip P , Brecosky J , Hammill K , et al. Insight in schizophrenia. Its relationship to acute psychopathology. J Nerv Ment Dis 1989; 177: 43–7. 2562850 10.1097/00005053-198901000-00007

[25] Guy W. ECDEU Assessment Manual for Psychopharmacology. U.S. Dept. of Health, Education, and Welfare; Public Health Service, Alcohol, Drug Abuse, and Mental health Administration, 1976.

[26] Heinrichs DW , Hanlon TE , Carpenter WT Jr . The Quality of Life Scale: an instrument for rating the schizophrenic deficit syndrome. Schizophr Bull 1984; 10: 388–98. 6474101 10.1093/schbul/10.3.388

[27] Stekhoven DJ , Bühlmann P. MissForest—non-parametric missing value imputation for mixed-type data. Bioinformatics 2012; 28: 112–8. 22039212 10.1093/bioinformatics/btr597

[28] Keefe RS , Bilder RM , Harvey PD , Davis SM , Palmer BW , Gold JM , et al. Baseline neurocognitive deficits in the CATIE schizophrenia trial. Neuropsychopharmacology 2006; 31: 2033–46. 16641947 10.1038/sj.npp.1301072

[29] Scutari M , Denis J-B. Bayesian Networks: With Examples in R. Chapman and Hall/CRC, 2021.

[30] Scutari M , Silander T. bnlearn: Bayesian network structure learning, parameter learning and inference. R package version 4.9.4. The Comprehensive R Archive Network, 2019.

[31] R Core Team. R: A Language and Environment for Statistical Computing. Version 4.3.2. R Foundation for Statistical Computing, 2024.

[32] Rapisarda A , Kraus M , Tan YW , Lam M , Eng GK , Lee J , et al. The continuous performance test, identical pairs: norms, reliability and performance in healthy controls and patients with schizophrenia in Singapore. Schizophr Res 2014; 156: 233–40. 24819191 10.1016/j.schres.2014.04.016

[33] Ruff R , Light R , Parker S , Levin H. Benton Controlled Oral Word Association Test: reliability and updated norms. Arch Clin Neuropsychol 1996; 11: 329–38. 14588937

[34] Gold JM , Carpenter C , Randolph C , Goldberg TE , Weinberger DR. Auditory working memory and Wisconsin Card Sorting Test performance in schizophrenia. Arch General Psychiatry 1997; 54: 159–65. 10.1001/archpsyc.1997.01830140071013 9040284

[35] Holm S. A simple sequentially rejective multiple test procedure. Scand J Stat 1979; 6: 65–70.

[36] McCleery A , Green MF , Hellemann GS , Baade LE , Gold JM , Keefe RSE , et al. Latent structure of cognition in schizophrenia: a confirmatory factor analysis of the MATRICS Consensus Cognitive Battery (MCCB). Psychol Med 2015; 45: 2657–66. 25916421 10.1017/S0033291715000641 PMC4523424

[37] Dickinson D , Ramsey ME , Gold JM. Overlooking the obvious: a meta-analytic comparison of digit symbol coding tasks and other cognitive measures in schizophrenia. Arch Gen Psychiatry 2007; 64: 532–42. 17485605 10.1001/archpsyc.64.5.532

[38] Eack SM , Newhill CE. Psychiatric symptoms and quality of life in schizophrenia: a meta-analysis. Schizophr Bull 2007; 33: 1225–37. 17204532 10.1093/schbul/sbl071 PMC2632363

[39] Watson P , Zhang J-P , Rizvi A , Tamaiev J , Birnbaum ML , Kane J. A meta-analysis of factors associated with quality of life in first episode psychosis. Schizophr Res 2018; 202: 26–36. 30005933 10.1016/j.schres.2018.07.013

[40] Carbon M , Correll CU. Thinking and acting beyond the positive: the role of the cognitive and negative symptoms in schizophrenia. CNS Spectr 2014; 19: 35–53. 10.1017/S1092852914000601 25403863

[41] Novick D , Montgomery W , Cheng Y , Moneta V , Haro J. Impact of negative symptoms on quality of life in patients with schizophrenia. Value Health 2015; 18: A836–7.

[42] Bouwmans C , de Sonneville C , Mulder CL , Hakkaart-van Roijen L. Employment and the associated impact on quality of life in people diagnosed with schizophrenia. Neuropsychiatr Dis Treat 2015; 11: 2125–42. 26316759 10.2147/NDT.S83546 PMC4547637

[43] Veerman SR , Schulte PF , De Haan L. Treatment for negative symptoms in schizophrenia: a comprehensive review. Drugs 2017; 77: 1423–59. 28776162 10.1007/s40265-017-0789-y

[44] Melillo A , Giordano GM , Caporusso E , Tomassini F , Perrottelli A , Giuliani L , et al. Association between cognitive deficits and negative symptoms: a systematic review of the literature. Eur Psychiatry 2023; 66: S1046–7.

[45] Rek-Owodziń K , Tyburski E , Plichta P , Waszczuk K , Bielecki M , Wietrzyński K , et al. The relationship between cognitive functions and psychopathological symptoms in first episode psychosis and chronic schizophrenia. J Clin Med 2022; 11: 2619. 35566742 10.3390/jcm11092619 PMC9102246

[46] Ventura J , Thames AD , Wood RC , Guzik LH , Hellemann GS. Disorganization and reality distortion in schizophrenia: a meta-analysis of the relationship between positive symptoms and neurocognitive deficits. Schizophr Res 2010; 121: 1–14. 20579855 10.1016/j.schres.2010.05.033 PMC3160271

[47] Amore M , Murri MB , Calcagno P , Rocca P , Rossi A , Aguglia E , et al. The association between insight and depressive symptoms in schizophrenia: undirected and Bayesian network analyses. Eur Psychiatry 2020; 63: 1–9. 10.1192/j.eurpsy.2020.45 PMC7358633 32372731

[48] Briganti G , Scutari M , McNally RJ. A tutorial on Bayesian networks for psychopathology researchers. Psychol Methods 2023; 28: 947–61. 35113632 10.1037/met0000479

[49] Santos B , González-Fraile E , Zabala A , Guillén V , Rueda JR , Ballesteros J. Cognitive improvement of acetylcholinesterase inhibitors in schizophrenia. J Psychopharmacol 2018; 32: 1155–66. 30324844 10.1177/0269881118805496

[50] Reddy LF , Horan WP , Jahshan C , Green MF. Cognitive remediation for schizophrenia: a review of recent findings. Curr Treat Options Psychiatry 2014; 1: 121–33.

[51] Bringmann LF , Elmer T , Epskamp S , Krause RW , Schoch D , Wichers M , et al. What do centrality measures measure in psychological networks? J Abnormal Psychol 2019; 128: 892–903. 10.1037/abn0000446 31318245

