# Supplementary material for: A symptom network approach to schizophrenia in the CATIE study: processing speed as the central cognitive impairment
Source: BJPsych Open. 2026 Jan 20;12(1):e42. doi: 10.1192/bjo.2025.10929 (PMC12835699; doi:10.1192/bjo.2025.10929)
Supplement: Buchwald et al. supplementary material [file S2056472425109290sup001.docx]

# Supplementary Materials

**Additional Analytic Strategies**

Networks are comprised of nodes (variables) and edges (associations between variables). One graphical network approach, BNs use directed acyclic graphs to decompose the joint probability distribution of the variables (20). Directed relationships encode the dependency structure among a set of variables. On a Windows PC, we used R version 4.3.2 (22) to analyse and implement the BNs in this study. To reconstruct relationship networks, we implemented a hybrid BN using the hill climbing method from the R package bnlearn (20). We used a hybrid BN. Hybrid BNs allow both discrete and continuous variables to be included in the model, using a mixture of multinomial distributions for the parameter estimation of the discrete variables and normal distributions for the parameter estimation of the continuous variables. The hill climbing algorithm was initialised with an empty network; edges are then iteratively deleted, added, or the edge direction is reversed to locally optimise a network score, which reflects a fit to the data and is penalised for complexity. We used the Bayesian Information Criterion (BIC) as a network score criterion. We implemented another score-based algorithm in bnlearn, Tabu, which led to the same solution in the network structure as the hill-climbing algorithm (20).

We also calculated a so-called averaged network based on reconstructed bootstrapped networks. More specifically, we created 1,000 bootstrapped versions of the data and implemented a hybrid BN on each of those versions of the data. We then estimated a significance threshold to select edges to be included in the averaged network. A significance threshold is a threshold that identifies the number of networks (from 1000) in which an edge must be present to be included in the averaged network. We optimised the calculation of the significance threshold using the averaged network function in bnlearn (20), and the optimal threshold was 491 of 1000 networks. Hence, if an edge was found in 491 bootstrapped networks or more, it was included in our averaged network model. The skeleton of the averaged network model structure is fitted to the complete data to build regressions, and the network parameters are then estimated. After reconstructing the BN on all data and averaged BN, we used the model structure to subject it to a structural equation model (SEM) and obtained the SEM linear model coefficients. We used the lavaan package (version 0.6-17) by Rosseel (23) to implement the SEM in R. The results for the averaged network can be found in the supplementary material section, except for the fit statistics. The network reconstructed on the complete data is now referred to as a BN, and the BN based on bootstrapped samples is named the averaged BN, two networks with different structures and parameters.

We computed several model fit statistics: Comparative fit index (CFI), where values above .95 are acceptable on a scale between 0 and 1; Tucker-Lewis index (TLI), where values above .95 are acceptable on a scale between 0 and 1; root mean square error of approximation (RMSEA), where values below approximately .06 are acceptable; BIC, with lower values indicating a better fit; and Akaike information criterion (AIC), with lower values indicating a better fit (24). BIC and AIC were extracted from the results of the BN, whereas CFI, TLI, and RMSEA were obtained from the results of the SEM. This is because the likelihood is not available as an output with mixed data types in the SEM of the lavaan package. We also conducted a hypothesis test of the model implied variance-covariance structure versus the observed variance-covariance structure to identify if the reconstructed model could plausibly have generated the data. Lastly, we extracted the model's standardised regression coefficients from the SEM, which scaled the variables to a mean of 0 and a standard deviation of 1, and the significance of each relationship in the regressions.

Following the reconstruction of the BN and averaged BN, we calculated centrality statistics for each of these. Centrality statistics arise from the complex interrelations between entities and are emergent network properties (25). The centrality statistics we calculated are closeness -the relationship between one node and all other nodes by taking into consideration the indirect connections from that node; betweenness -the importance of the node in relationship to all other pairs of nodes in the network; and degree -the number of neighbours a node has in the network (26). Closeness and betweenness are standardised, and values for degree are unstandardised in this study. It is not possible to estimate closeness for nodes without outgoing edges. Next, we queried the conditional probability between the nodes representing cognition and predictors of QOL and QOL, as explained in Briganti, Scutari (27). These were used to infer how an event, such as scoring above the median on processing speed, is associated with the probability of other nodes, such as scoring below the median on working memory (27). The R code used to conduct the analysis can be found in the Data Availability Statement section.

**Table S1**

*Adjusted p Values for Model 1*

| Parent | Child | p (orginal) | p (adjusted) |
| --- | --- | --- | --- |
| Chi Square model fit |  | <.001 | <.001 |
| Age | Marital.Status | <.001 | <.001 |
| Age | Education | <.001 | .002 |
| Age | DAI | <.001 | <.001 |
| Age | CGI.Drug.Use | <.001 | <.001 |
| Race | CGI.Drug.Use | .001 | .017 |
| Marital.Status | Sex | <.001 | <.001 |
| Age | MATRICS.Processing.Speed | <.001 | <.001 |
| Education | MATRICS.Processing.Speed | <.001 | <.001 |
| Sex | CDSS | .004 | .040 |
| DAI | CDSS | <.001 | <.001 |
| CGI.Drug.Use | CDSS | <.001 | <.001 |
| Age | MATRICS.Reasoning | <.001 | <.001 |
| MATRICS.Processing.Speed | MATRICS.Reasoning | <.001 | <.001 |
| Sex | CGI.Alcohol.Use | <.001 | <.001 |
| CGI.Drug.Use | CGI.Alcohol.Use | <.001 | <.001 |
| MATRICS.Processing.Speed | MATRICS.Memory | <.001 | <.001 |
| MATRICS.Reasoning | MATRICS.Memory | <.001 | <.001 |
| MATRICS.Processing.Speed | MATRICS.Vigil | <.001 | <.001 |
| MATRICS.Memory | MATRICS.Vigil | <.001 | <.001 |
| CDSS | PANSS.General | <.001 | <.001 |
| DAI | PANSS.General | <.001 | <.001 |
| MATRICS.Memory | PANSS.General | <.001 | <.001 |
| CDSS | PANSS.Negative | <.001 | <.001 |
| MATRICS.Processing.Speed | PANSS.Negative | <.001 | <.001 |
| PANSS.General | PANSS.Negative | <.001 | <.001 |
| CGI.Alcohol.Use | PANSS.Negative | .231 | 1 |
| DAI | PANSS.Positive | <.001 | .001 |
| PANSS.General | PANSS.Positive | <.001 | <.001 |
| PANSS.Negative | PANSS.Positive | .002 | .022 |
| CGI.Drug.Use | PANSS.Positive | <.001 | .001 |
| MATRICS.Memory | CGI.Severity | .005 | .048 |
| PANSS.General | CGI.Severity | .001 | .010 |
| PANSS.Negative | CGI.Severity | <.001 | <.001 |
| PANSS.Positive | CGI.Severity | <.001 | <.001 |
| Employment | QOL | <.001 | <.001 |
| CDSS | QOL | <.001 | .003 |
| DAI | QOL | <.001 | <.001 |
| MATRICS.Processing.Speed | QOL | <.001 | <.001 |
| PANSS.Negative | QOL | <.001 | <.001 |
| CGI.Severity | QOL | <.001 | <.001 |
| CDSS | ITAQ | <.001 | <.001 |
| DAI | ITAQ | <.001 | <.001 |
| PANSS.General | ITAQ | <.001 | <.001 |
| QOL | ITAQ | <.001 | <.001 |
| MATRICS.Processing.Speed | MATRICS.Verbal | <.001 | <.001 |
| MATRICS.Memory | MATRICS.Verbal | <.001 | <.001 |
| QOL | MATRICS.Verbal | <.001 | <.001 |

**Figure S1**

*Averaged Bayesian Network*

*
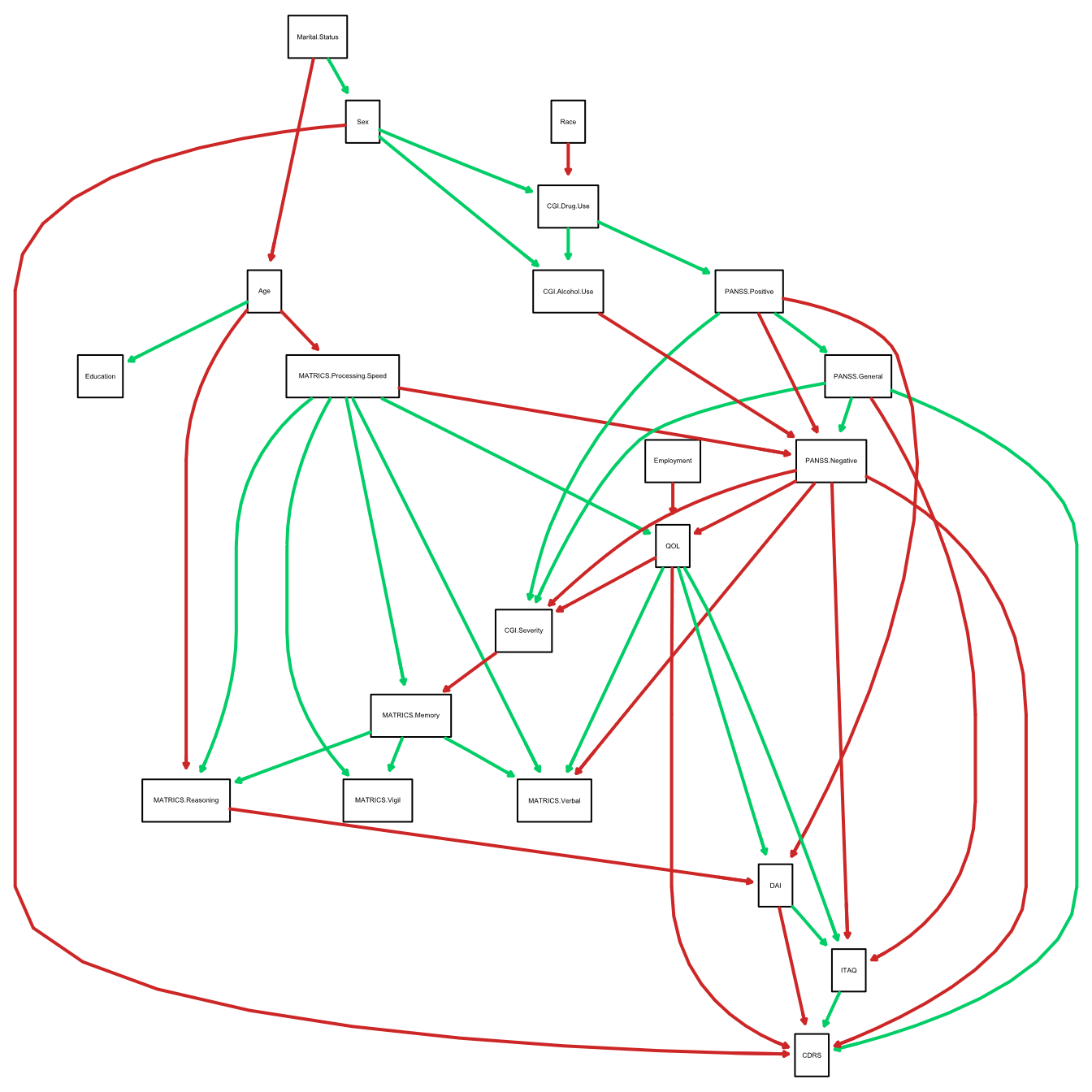
*

**Figure S2**

*Structural Equation Model of Averaged Bayesian Network*

*
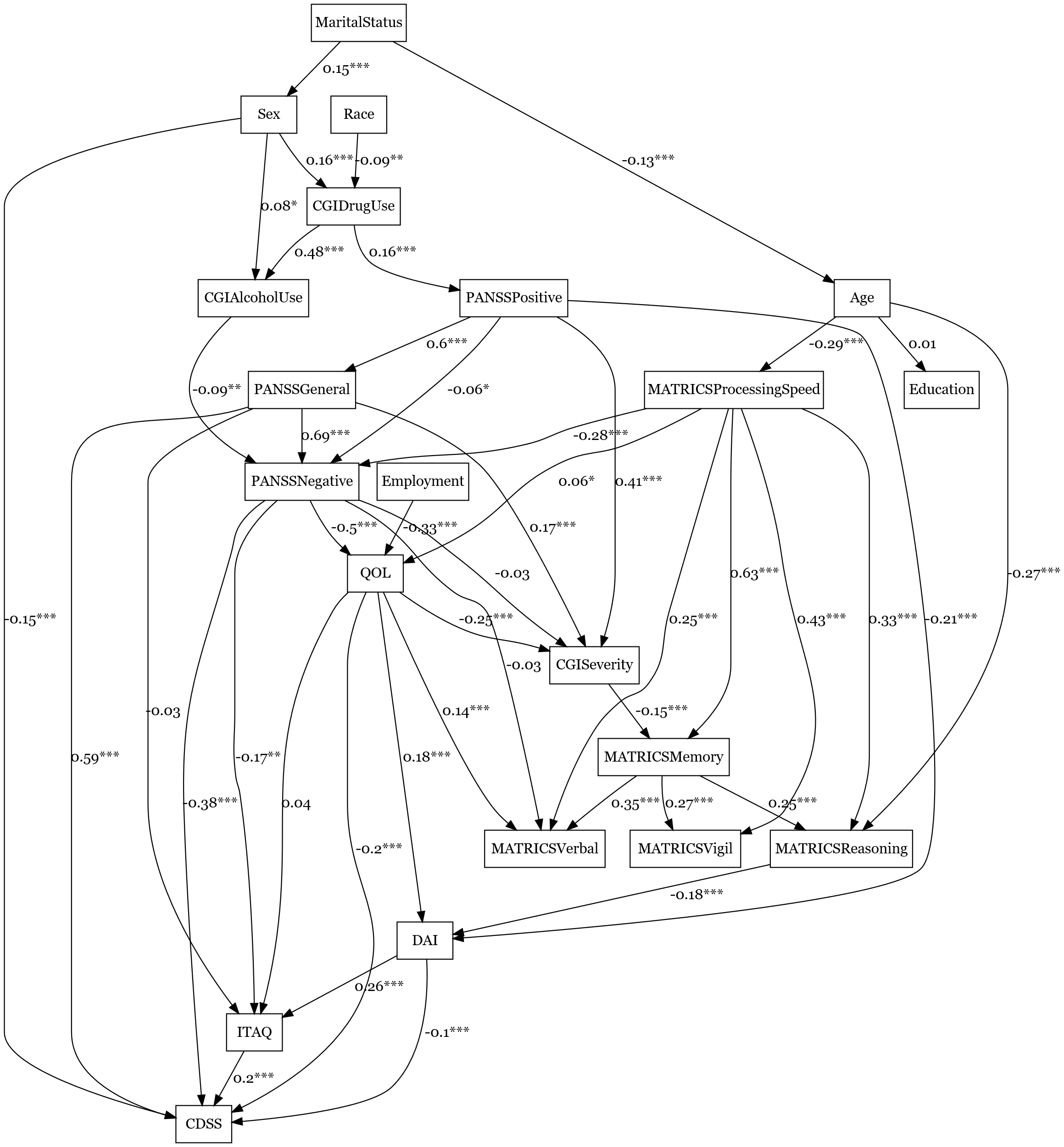
*

**Figure S3**

*Centrality Statistics of the Averaged Bayesian Network*

|  |  |  |
| --- | --- | --- |

*^Note: Standardised values given for closeness and betweenness; 1 = Age; 2 = Sex; 3 = Race; 4 = Marital Status; 5 = Education ;6 = Employment; 7 = CDSS total; 8 = DAI total; 9 = ITAQ total; 10 = MATRICS verbal; 11 = MATRICS Vigilance; 12 = MATRICS processing speed; 13 = MATRICS reasoning; 14 = MATRICS working memory; 15 = PANSS general; 16 = PANSS negative; 17 = PANSS positive; 18 =CGI Drug Use; 19 =CGI Alcohol use; 20 = CGI Severity; 21 = QOL.^*

**Table S2**

*Adjusted p Values for Model 2*

| Parent | Child | p (orginal) | p (adjusted) |
| --- | --- | --- | --- |
| Chi Square model fit |  | <.001 | <.001 |
| Marital.Status | Age | <.001 | .008 |
| Marital.Status | Sex | <.001 | .001 |
| Age | Education | .908 | 1 |
| Age | MATRICS.Processing.Speed | <.001 | <.001 |
| Sex | CGI.Drug.Use | <.001 | <.001 |
| Race | CGI.Drug.Use | .001 | .017 |
| CGI.Drug.Use | PANSS.Positive | <.001 | <.001 |
| Sex | CGI.Alcohol.Use | .022 | .174 |
| CGI.Drug.Use | CGI.Alcohol.Use | <.001 | <.001 |
| PANSS.Positive | PANSS.General | <.001 | <.001 |
| MATRICS.Processing.Speed | PANSS.Negative | <.001 | <.001 |
| PANSS.General | PANSS.Negative | <.001 | <.001 |
| PANSS.Positive | PANSS.Negative | .036 | .255 |
| CGI.Alcohol.Use | PANSS.Negative | .001 | .017 |
| Employment | QOL | <.001 | <.001 |
| MATRICS.Processing.Speed | QOL | .017 | .153 |
| PANSS.Negative | QOL | <.001 | <.001 |
| PANSS.General | CGI.Severity | <.001 | .006 |
| PANSS.Negative | CGI.Severity | .555 | 1 |
| PANSS.Positive | CGI.Severity | <.001 | <.001 |
| QOL | CGI.Severity | <.001 | <.001 |
| MATRICS.Processing.Speed | MATRICS.Memory | <.001 | <.001 |
| CGI.Severity | MATRICS.Memory | <.001 | <.001 |
| MATRICS.Processing.Speed | MATRICS.Verbal | <.001 | <.001 |
| MATRICS.Memory | MATRICS.Verbal | <.001 | <.001 |
| PANSS.Negative | MATRICS.Verbal | .431 | 1 |
| QOL | MATRICS.Verbal | <.001 | <.001 |
| MATRICS.Processing.Speed | MATRICS.Vigil | <.001 | <.001 |
| MATRICS.Memory | MATRICS.Vigil | <.001 | <.001 |
| Age | MATRICS.Reasoning | <.001 | <.001 |
| MATRICS.Processing.Speed | MATRICS.Reasoning | <.001 | <.001 |
| MATRICS.Memory | MATRICS.Reasoning | <.001 | <.001 |
| MATRICS.Reasoning | DAI | <.001 | <.001 |
| PANSS.Positive | DAI | <.001 | <.001 |
| QOL | DAI | <.001 | <.001 |
| DAI | ITAQ | <.001 | <.001 |
| PANSS.General | ITAQ | .549 | 1 |
| PANSS.Negative | ITAQ | .002 | .025 |
| QOL | ITAQ | .173 | 1 |
| Sex | CDSS | <.001 | <.001 |
| DAI | CDSS | <.001 | .003 |
| ITAQ | CDSS | <.001 | <.001 |
| PANSS.General | CDSS | <.001 | <.001 |
| PANSS.Negative | CDSS | <.001 | <.001 |
| QOL | CDSS | <.001 | <.001 |
